# Bone scan positivity in non-metastatic, castrate-resistant prostate cancer: external validation study

**DOI:** 10.1590/S1677-5538.IBJU.2019.0225

**Published:** 2020-01-13

**Authors:** Ashley W. Johnston, Thomas A. Longo, Leah Gerber Davis, Daniel Zapata, Stephen J. Freedland, Jonathan C. Routh

**Affiliations:** 1 Division of Urologic Surgery, Duke University Medical Center, Durham, NC, USA; 2 Division of Urology, Cedars-Sinai Medical Center, Los Angeles, CA, USA

**Keywords:** Prostate, Medical Oncology, Radiology, Prostate-Specific Antigen

## Abstract

**Introduction:**

Tables predicting the probability of a positive bone scan in men with non-metastatic, castrate-resistant prostate cancer have recently been reported. We performed an external validation study of these bone scan positivity tables.

**Materials and Methods:**

We performed a retrospective cohort study of patients seen at a tertiary care medical center (1996-2012) to select patients with non-metastatic, castrate-resistant prostate cancer. Abstracted data included demographic, anthropometric, and disease-specific data such as patient race, BMI, PSA kinetics, and primary treatment. Primary outcome was metastasis on bone scan. Multivariable logistic regression was performed using generalized estimating equations to adjust for repeated measures. Risk table performance was assessed using ROC curves.

**Results:**

We identified 6.509 patients with prostate cancer who had received hormonal therapy with a post-hormonal therapy PSA ≥2ng/mL, 363 of whom had non-metastatic, castrate-resistant prostate cancer. Of these, 187 patients (356 bone scans) had calculable PSA kinetics and ≥1 bone scan. Median follow-up after castrate-resistant prostate cancer diagnosis was 32 months (IQR: 19-48). There were 227 (64%) negative and 129 (36%) positive bone scans. On multivariable analysis, higher PSA at castrate-resistant prostate cancer (4.67 vs. 4.4ng/mL, OR=0.57, P=0.02), shorter time from castrate-resistant prostate cancer to scan (7.9 vs. 14.6 months, OR=0.97, P=0.006) and higher PSA at scan (OR=2.91, P <0.0001) were significantly predictive of bone scan positivity. The AUC of the previously published risk tables for predicting scan positivity was 0.72.

**Conclusion:**

Previously published risk tables predicted bone scan positivity in men with non-metastatic, castrate-resistant prostate cancer with reasonable accuracy.

## INTRODUCTION

Non-metastatic castrate resistant prostate cancer (M0 CRPC) carries a large burden of disease. Patients are largely asymptomatic in this disease state, thus, it is an important clinical goal to prevent further progression to metastatic disease. Within 2 years, 46% of M0 CRPC patients develop metastases with 33% representing bony metastases (1, 2). The progression from M0 to metastatic (M1) CRPC is a seminal event affecting patient and provider decision-making (3). While two drugs are approved for M0 CRPC, apalutamide and enzalutamide (4), there are a multitude of options in the treatment of M1 CRPC including abiraterone, sipuleucel-T, and denosumab (5–7), and perhaps in the future, combination therapies that are not available for M0 CRPC. In addition, given differing outcomes of M0 and M1 CRPC patients, positive imaging has a strong prognostic value. Thus, the separation of M0 CRPC from M1 CRPC remains critically important.

The ability to detect progress is not well understood given the heterogeneity of this disease stage. Thus, the challenge is to identify clinical factors that would appropriately trigger ordering a bone scan in CRPC patients. Prior studies have shown that among men with presumed M0 CRPC, upwards of one-third of patients actually have M1 CRPC when subjected to a metastatic work up (8). Unfortunately, there are studies showing that bone scans may be either over- or underused in this common clinical scenario (9, 10). In order to identify men at high-risk for a positive bone scan, data including PSA levels and kinetics from 312 men were used to develop prediction tables. Frequent PSA levels are affordable and the standard of care making them an attractive trigger. The original study developing these prediction tables was conducted using data from two Veterans Affairs (VA) medical centers (11), and the subsequent validation study was performed in 3 additional VA medical centers involving 281 men (12). In the current climate of scientific rigor, validation and replication are becoming increasingly important. We therefore performed a validation study at a large tertiary academic medical center. Our objective was to generalize the prior studies into a population of non-VA patients.

## MATERIALS AND METHODS

### Study Population

**Figure 1:**
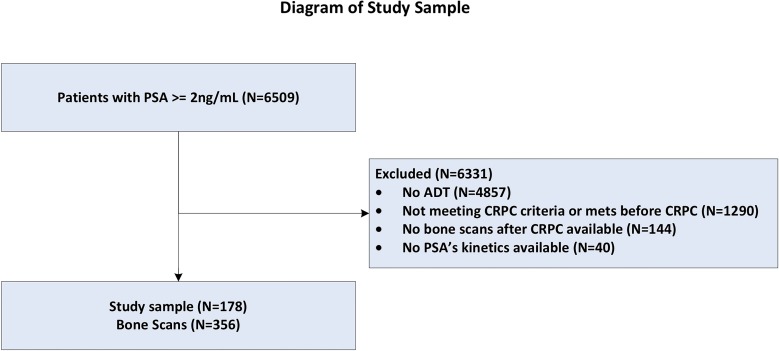
CONSORT diagram of the study population.

After receiving Institutional Review Board approval, patient records from our institution were reviewed. Based on electronic billing records augmented by a detailed chart review, we identified 6.509 patients with prostate cancer, who had received at least 1 dose of androgen deprivation therapy (ADT), and who had a PSA value ≥2.0ng/mL after the receipt of ADT. Medical records were manually screened to select patients with documented M0 CRPC. Of these 6.509 patients, 4.847 were excluded for not having continuous ADT. Another 1.290 were excluded for not having CRPC or having documented metastatic disease prior to CRPC diagnosis. Thus, 363 patients had documented M0 CRPC as defined by the Prostate Cancer Working Group 2 definition: a 25% or greater rise in PSA and an absolute increase of ≥ 2.0ng/mL from the post-ADT PSA nadir while receiving continuous ADT or after orchiectomy (11). Our final cohort was limited to 187 patients who had at least one bone scan after CRPC diagnosis as well as available PSA kinetics (Figure-1). The final database included information on race, age at the time of bone scans, height, weight, PSA, diagnosis date, primary treatments, clinical and pathological characteristics, PSA values, bone scan results, as well as follow-up. Bone scan results were coded as positive or negative based on the radiology reports and subsequent imaging (if equivocal). Equivocal scans were considered negative unless proven positive by a second imaging modality or biopsy. Patients were followed up to their first positive bone scan. Once a patient was documented as having bony metastasis no further scans were reviewed.

#### Statistical analysis

PSA doubling time (PSADT) was calculated by the natural log of two divided by the slope of the linear regression of the natural log of the PSA over time in months. PSA's from the time of CRPC diagnosis or two years prior to the scan (whichever was closer to the scan) up until the scan date were included in PSADT calculations. To calculate a PSADT, ≥ 2 PSA's over at least three months were required. If PSADT was declining or at >120 months a PSADT of 120 was assigned for ease of analysis.

Baseline characteristics (at time of CRPC index date) were summarized using median, and first and third quartiles for continuous variables and frequency and percentages for categorical variables. Univariable models were fit with the following variables: age at CRPC index date (years), year of scan, race (black or non-black), biopsy pathological grade group (1–5), primary treatment (radical prostatectomy ± radiation, radiation only, other/unknown), time from ADT to CRPC (months), PSA at CRPC index date (ng/mL), time from CRPC to scan (months), PSA at time of scan (ng/mL) and PSADT at the time of scan (months). PSA at the time of scan was log transformed. Age at CRPC and year of scan were centered by their mean value to eliminate multicollinearity. These characteristics were compared between the subsets of negative and positive bone scans. To account for multiple bone scans per patient (repeated measures), P-values were calculated using generalized estimating equations (GEE) using a logit link, autoregressive correlation structure, and type 3 estimation.

Multivariable analyses were also performed using GEE methods including all covariates from the univariable analysis. Pre-scan PSA and PSADT were somewhat correlated (Spear-man=-0.11, P-value 0.04), but not collinear and thus both variables were retained in the analysis.

To assess the performance of the Moreira et al. risk table to predict bone scan positivity among men with M0 CRPC in our cohort, PSA levels were divided into four groups (<5, 5- <15, 15- <50, ≥50) and PSADT was divided into four groups (≥ 15, ≥ 9-15, ≥ 3-9, and <3) based on previously identified cut points (11). A calibration curve was performed to show the performance of the risk table. ROC curves were constructed and AUC calculated to assess the predictive accuracy of the Moreira et al. model compared to our cohort.

All statistical analyses were performed using SAS version 9.4 and R version 3.4.1.

## RESULTS

Among the 187 patients with PSA kinetic data, 356 bone scans were performed from CRPC diagnosis until the first positive scan. Median age of CRPC diagnosis was 72 years (IQR: 64-79) and median year of diagnosis was 2008 (IQR: 2004-2010) ranging from 1997 to 2012. Median follow-up after CRPC diagnosis was 32 months (IQR: 19-48) (Table-1). Median number of bone scans per patient was 1 (IQR: 1-2). The maximum number of scans for one individual was 12. Half of the subjects (51%) had only one bone scan. A positive scan for metastasis was noted in 73%; of those with metastasis, 69% were diagnosed on their first scan after CRPC diagnosis. Of those without a positive scan, 22% had more than 5 scans. No subjects with a positive scan had more than 5 scans.

**Table 1:** Baseline Patient Characteristics.

Variables		N=187
Number of Bone Scans, median (Q1, Q3)		1 (1, 2)
Age at CRPC (years), median (Q1, Q3)		72 (64, 79)
Year of CRPC Diagnosis, median (Q1, Q3)		2008 (2004, 2010)
**Race, N (%)**		
	Non-black	135 (72)
	Black	52 (28)
**Biopsy Gleason Score, N (%)**		
	2-6	35 (19)
	7	43 (23)
	8-10	48 (26)
Unknown/No Biopsy		61 (32)
**Primary Treatment, N (%)**		
	None/Unknown/Other	70 (37)
	RP ± Radiation	80 (43)
	Radiation Alone	37 (20)
Time from ADT to CRPC (months), median (Q1, Q3)		39 (18, 65)
PSA at Diagnosis (ng/mL), median (Q1, Q3)		12.2 (7.0, 25.9)
PSA at CRPC (ng/mL), median (Q1, Q3)		4.6 (2.8, 10.1)
Total Follow-up (months), Median (Q1, Q3)		32 (19, 48)

Baseline characteristics stratified by positive and negative scan results are shown in Table-2. Patients are repeated in these counts if they had more than one scan. There were 227 (64%) negative and 129 (36%) positive bone scans. Positive bone scans were associated with tendency to have radical prostatectomy+/-radiation as primary treatment (46% vs. 33%, OR=2.32, P=0.016) and greater pre-scan PSA value (27.3 vs. 7.1, OR=1.97, P <0.0001), compared to negative scans (Table 2 and 3). Younger age (70 vs. 73 years, OR=0.97, P=0.019), and shorter pre-scan PSADT (5.9 vs. 11.3, OR=0.68, P=0.0002) were statistically significantly related to scan positivity. There were no associations between bone scan positivity and year of bone scan, race, biopsy pathological grade group, time from ADT to CRPC, or time from CRPC to scan (all p-values >0.08).

**Table 2:** Baseline Patient Characteristics.

Variables		Negative Bone Scan N=227	Positive Bone Scan N=129	P*
Age at CRPC (years), median (Q1, Q3)		73 (67, 79)	70 (63, 77)	0.019
Year of Scan, median (Q1, Q3)		2009 (2007, 2011)	2009 (2006, 2010)	0.768
**Race, N (%)**				
	Non-black	158 (70)	94 (73)	0.655
	Black	69 (30)	35 (27)	
Biopsy Gleason Score, N (%)				0.68
	2-6	51 (22)	24 (19)	
	7	55 (24)	28 (22)	
	8-10	53 (23)	37 (29)	
Unknown/No Biopsy		68 (30)	40 (31)	
Primary Treatment, N (%)				**0.016**
	None/Unknown/Other	118 (52)	41 (32)	
	RP ± Radiation	74 (33)	59 (46)	
Radiation Alone		35 (15)	29 (22)	
Time from ADT to CRPC (months), median (Q1, Q3)		41 (28, 65)	34 (17, 60)	0.159
PSA at CRPC (ng/mL), median (Q1, Q3) ¥		4.4 (2.8, 9.13)	4.67 (2.79, 10.88)	0.082
Time from CRPC to scan (months), median (Q1, Q3)		14.59 (5.05, 29.27)	7.89 (2.92, 21.35)	0.766
Pre-scan PSA (ng/mL), median (Q1, Q3) ¥		7.1 (2.9, 19.86)	27.25 (9,79.6)	<0.0001
Pre-scan PSADT (ng/mL), median (Q1, Q3) ¥		11.34 (6.00, 44.66)	5.91 (3.41, 13.42)	0.0002

¥ = Log-transformed variable was used in this analysis

**Table 3:** Predictors of Bone Scan Positivity.

Variables		Univariate Results		Multivariate Results		
		OR (95% CI)	P	OR (95% CI)	P
Age at CRPC (years), median (Q1, Q3)		0.97 (0.94-0.99)	0.019	0.99 (0.96-1.03)	0.697
Year of Scan, median (Q1, Q3)		0.99 (0.92-1.06)	0.768	1.06 (0.97-1.16)	0.204
**Race, N (%)**			**0.655**		0.738
	Non-black	ref		ref	
	Black	0.87 (0.47-1.6)		1.13 (0.54-2.38)	
**Biopsy Gleason Score, N (%)**			0.68		0.632
	2-6	ref		ref	
	7	1.04 (0.46-2.34)		1.25 (0.50-3.1)	
	8-10	1.54 (0.69-3.46)		1.60 (0.66-3.92)	
Unknown/No Biopsy		1.18 (0.55-2.54)		1.69 (0.72-3.97)	
**Primary Treatment, N (%)**			0.016		0.12
	None/Unknown/Other	ref		ref	
	Radiation Alone	2.24 (1.15, 4.35)		1.85 (0.78-4.42)	
RP ± Radiation		2.32 (1.25, 4.32)		2.37 (1.02-5.49)	
Time from ADT to CRPC (months), median (Q1, Q3)		0.99 (0.99-1.00)	0.159	1.00 (0.99-1.01)	0.729
PSA at CRPC (ng/mL), median (Q1, Q3) ¥		1.26 (0.98-1.61)	0.082	0.57 (0.37-0.87)	0.016
Time from CRPC to scan (months), median (Q1, Q3)		1.00 (0.98-1.01)	0.766	0.97 (0.95-0.99)	0.006
Pre-scan PSA (ng/mL), median (Q1, Q3) ¥		1.97 (1.65-2.35)	<0.0001	2.91 (2.17-3.92)	<0.0001
Pre-scan PSADT (ng/mL), median (Q1, Q3) ¥		0.68 (0.57-0.82)	0.0002	1.12 (0.87-1.46)	0.386

¥ = Log-transformed variable was used in this analysis

On multivariable analysis, higher PSA at CRPC (4.67 vs. 4.4ng/mL, OR=0.57, P=0.02), shorter time from CRPC to scan (7.9 vs. 14.6 months, OR=0.97, P=0.006), and higher pre-scan PSA (OR=2.91, P <0.0001) were significantly predictive of bone scan positivity.

**Figure 2:**
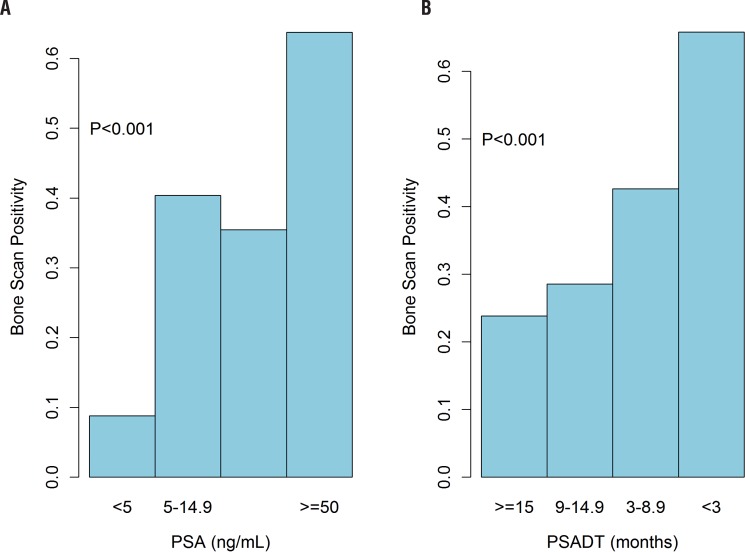
Bone scan positivity by PSA (A) and by PSA doubling time (B).

**Figure 3:**
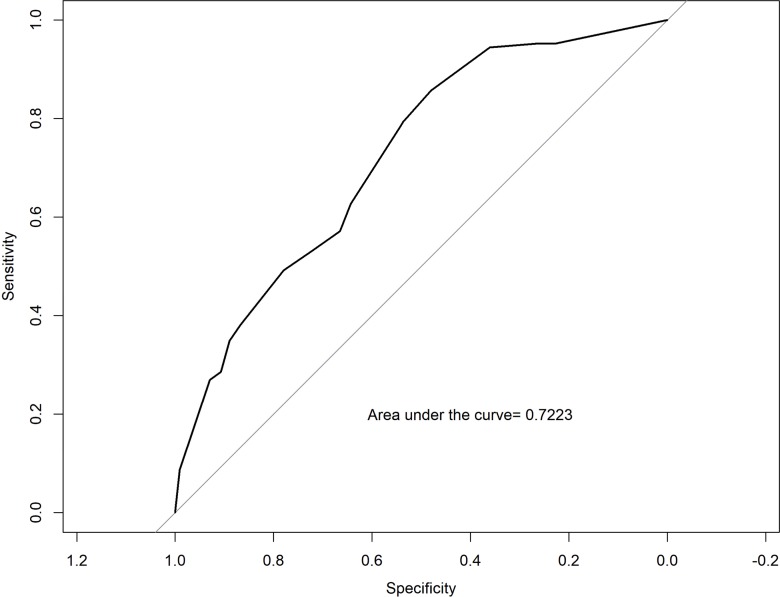
Receiver-Operator Characteristics Curve of the Moreira Tables Predicting Bone Scan Positivity.

**Figure 4:**
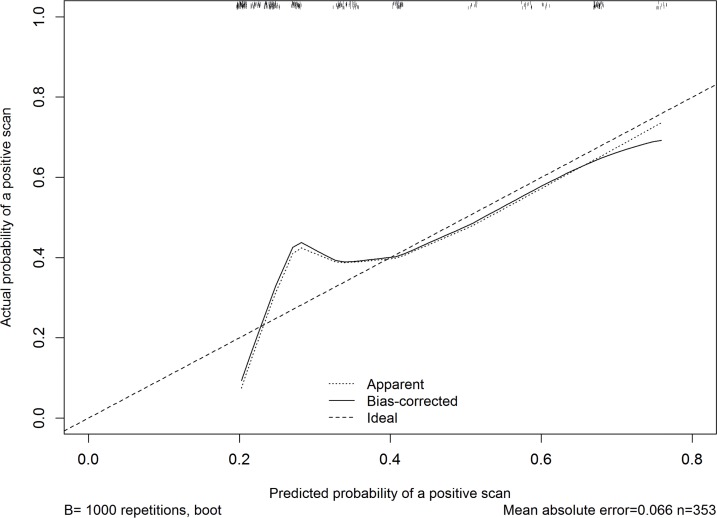
Calibration curve demonstrating general agreement between predicted and actual probability of a positive scan.

In the analysis by PSA groups (Chi-square), the scan positivity was 8.8%, 40.4%, 35.4%, and 63.8% for men with PSA <5, 5- <15, 15- <50, ≥ 50ng/mL, respectively (Figure-2A, P<0.0001). Men with PSADT ≥ 15, ≥ 9-15, ≥ 3-9, and <3months had scan positivity of 23.8%, 28.6%, 42.6%, and 65.8%, respectively (Figure-2B, P <0.0001). The AUC of the Moreira et al. table in predicting rates of positive scan was 0.72 (Figure-3). A calibration curve demonstrated good agreement between predicted and actual probability of a positive scan, except if the estimated probability of a positive scan was <40%, when the model mostly underestimated the probability of a positive result (Tables 4 and 5, Figure-4).

**Table 4:** Moreira et al. (11) Predicted risk of positive scan by PSA and PSADT groups.

PSADT (months)	PSA (ng/mL)			
	<5	5-14.9	15-49.9	≥ 50
≥ 15	6 (4-8)	11 (9-14)	22 (18-28)	47(40-54)
9-14.9	6 (4-10)	12 (10-14)	24 (22-26)	49 (46-52)
3-8.9	8 (5-14)	16 (13-18)	30 (27-33)	57 (53-60)
<3	12 (8-19)	22 (19-25)	40 (37-42)	67 (64-69)

**Table 5:** Predicted risk of positive scan by PSA and PSADT groups.

PSADT (months)	PSA (ng/mL)			
	<5	5-14.9	15-49.9	≥ 50
≥ 15	10 (6/58)	29 (11/38)	28 (5/18)	67 (8/12)
9-14.9	0 (0/9)	38 (8/21)	28 (5/18)	29 (2/7)
3-8.9	5 (1/22)	47 (20/43)	41 (14/34)	62 (23/37)
<3	50 (1/2)	58 (7/12)	44 (4/9)	85 (11/13)

## DISCUSSION

Approximately 90% of men with CRPC will develop bone metastases and the burden of disease correlates directly with survival (13). While the AUA, EAU, and NCCN guidelines provide clear recommendations for initial screening with a bone scan at the time of diagnosis in high-risk men, they do not provide specific recommendations for metastatic screening of asymptomatic CRPC patients (14–16). The algorithm we sought to validate in this study takes advantage of PSA and PSADT to create a prediction tool to predict the risk of a positive bone scan among men with M0 CRPC and fill a gap in existing guidelines. Prediction tools tend to outperform human experts (17), and the algorithm validated in this study serves to augment the existing guidelines and avoid unnecessary bone scans, while still identifying metastatic disease.

The AUC is a measure of the accuracy of a test. Our goal was to test how accurately the Moreira et al. tables can distinguish between the group of men who will have a positive or negative bone scan. An AUC of 0.72 represents a good test for clinical applications. Our study validates this tool in a cohort with both a large proportion (28%) of African Americans and further expands its use beyond the VA to the general population.

Many of the large prostate cancer trials are predominantly comprised of European men or men of European descent. The development and validation of the Moreira et al. tables included a large portion of African American men. SEER data has demonstrated that African American men have a 64% higher incidence of prostate cancer than white men (18). They also tend to present with higher grade and stage tumors, leading to a 2.4-fold increase in prostate cancer mortality when compared to white men (19). Prior to published guidelines in the mid-90s, African American men were less likely to undergo appropriate imaging, but this difference was resolved with more contemporary studies (20). Within this cohort, the median baseline PSA was higher (<0.001), and the PSADT was shorter (p <0.01) for African Americans with prostate cancer compared to white men (21). There is evidence that even after adjusting for differences in social determinants of health, a higher mortality rate from prostate cancer still persists for African American compared to white men (22). North Carolina's population is 21% African American; the incidence of prostate cancer in this population is 216.5 per 100.000 (disparity ratio of 1.7) and the mortality rate is 44.2 per 100.000 (disparity ratio of 2.5) (23). Valid prediction tools for the African American population are thus understudied but essential.

In addition to including a large portion of African Americans, this study population moves beyond the VA and into the broader community. The patient population from the VA have contributed greatly to medicine, and there are several seminal manuscripts in urology, particularly prostate cancer, that derive from this cohort (24). Prostate cancer treatment is similar between the VA and general community (25). However, VA patients are a unique subset of the American public, and have a distinct demographic distribution. VA patients tend to be older, sicker, and of lower socioeconomic status than the US population (26). Patients at the VA are diagnosed with commonly occurring cancers at earlier stages, relative to the general population (25). Prostate cancer accounts for roughly 33% of cancer diagnoses among men within the VA, and only 25% in the general population (26.). Validation in a tertiary hospital makes the results more generalizable.

Our results showed that a positive scan was more likely to be associated with a history of radical prostatectomy. This would suggest that in the setting of extirpative surgery, a rising PSA is more likely to come from a metastasis than a local recurrence, and this has been seen before, even in the setting of node positive disease (27, 28).

This study has several limitations. It is a retrospective study in a single institution, and as such these data subject to secular trends, practice pattern variation, and/or care differences that may limit the generalizability of its findings. Furthermore, this institution is a tertiary medical center, and thus these findings (particularly the severity of disease in this population) may not be truly representative of the broader prostate cancer community. Also, it is possible that with a larger study, other variables, such as Gleason score, may correlate with positive imaging that could further improve risk stratification. Nonetheless, the facts that these tables are accurate in both a VA setting and a tertiary care facility gives some credence that these can be widely applied, though ideally validation in the community would be needed. While a 10% duplicate data entry system and rigorous data quality checks were used throughout data abstraction and analysis, these data may also be subject to information or miscoding bias, as with any retrospective analysis. Finally, while they are clinically useful, the accuracy of the tables may not be ideal. As such, future research should focus on additional biomarkers of metastases to further aid in risk stratification for this important group of prostate cancer patients.

## CONCLUSIONS

In conclusion, among men with M0 CRPC seen at a tertiary care academic medical center, the Moreira et al. risk tables predicted bone scan positivity with reasonable accuracy and significant improvement over PSA and PSA kinetics alone. The tables have now been externally validated using multiple datasets and appear to be generalizable to the larger medical community.
